# CABG at a Crossroads: Reinventing the Distal Anastomosis

**DOI:** 10.1093/icvts/ivaf210

**Published:** 2025-09-28

**Authors:** Willem J L Suyker, John D Puskas

**Affiliations:** Division of Heart and Lungs, Cardiothoracic Surgery, UMC Utrecht, Utrecht, The Netherlands; Division of Cardiothoracic Surgery, Emory University School of Medicine, Atlanta, Georgia, United States

Coronary artery bypass grafting (CABG) remains the most effective strategy for complex multivessel disease—but at the cost of significant invasiveness. It is sobering that since myocardial stabilizers and off-pump surgery were introduced in the late 1990s, no major procedural innovation has gained broad traction. Even off-pump techniques, despite physiological appeal, have stalled at a modest 15-18% global uptake. Meanwhile, other surgical fields—urology, gynecology, and abdominal surgery—have widely embraced minimally invasive and robotic methods, with substantial patient benefits.

Coronary surgery remains largely open-chested, even off-pump. The technically demanding distal anastomosis continues to resist simplification and is the main barrier to truly minimally invasive CABG.

Nearly a century of vascular surgical wisdom—rooted in Alexis Carrel’s principles—continues to guide anastomosis construction: minimal trauma, full endothelial coverage, and a widely patent geometry. The process involves three deceptively simple steps: creating an opening, aligning vessel edges, and joining them—traditionally with sutures. When done precisely, this ensures durable patency. In practice, coronary targets are small and unforgiving: vessel diameters typically range from 1.25 to 2.5 mm, and sub-millimetric accuracy is essential. Suturing is also inherently inefficient, as each vessel wall must be manipulated sequentially. These factors make conventional suturing poorly suited for minimally invasive or endoscopic use.

Despite these challenges, pioneers like Sam Balkhy have shown that with passion, persistence, and patience, robot-assisted suturing can match open results in total endoscopic CABG (TECAB).^1^ His series demonstrated clear clinical benefits, including next-day discharge and rapid recovery. Yet complex workflows and steep learning curves have limited broader uptake—underscoring the need for breakthrough solutions. Replacing hand-suturing with sutureless, (semi-)automated connector technologies could redefine the field, as staplers once did for bowel surgery. Past experience has made this path controversial, however.

The idea of sutureless connectors for coronary bypass is not new. In 1967, Kolesov and Demikhov performed the first known clinical case using a vascular stapler—the VCA-4—to connect the internal mammary artery to the LAD on the beating heart via an intercostal approach.^2^ Technically intriguing and conceptually ahead of its time, the method remained obscure. Its end-to-end geometry and time-consuming deployment limited utility, while its Soviet origin likely contributed to a lack of international attention.

Renewed interest in sutureless technology emerged only decades later, catalyzed by the rise of off-pump surgery and intended to support its broader uptake. Initially, these systems aimed to automate the proximal anastomosis, typically between the saphenous vein graft and the aorta. Devices such as the Symmetry (St Jude Medical, St Paul, MN, USA), CorLink (Bypass Ltd, Herzelia, Israel), and PAS-Port (Cardica Inc, Redwood, CA, USA) entered clinical trials and saw limited uptake. Enthusiastically embraced by early adopters, reports of disappointing outcomes—particularly with the Symmetry device—soon cast doubt on their viability.

Distal anastomosis devices followed shortly after, with 5 concepts reaching clinical evaluation: the SJM distal connector (St Jude Medical, St Paul, MN, USA), AADD (Bypass Ltd, Herzelia, Israel), MVP (Ventrica Inc, Fremont, CA, USA), the Converge Coupler (Converge Medical, Sunnyvale, CA, USA), and the C-Port (Cardica Inc, Redwood, CA, USA). Patency outcomes—arguably the most critical performance measure—were often unconvincing due to either suboptimal results or clear target vessel selection bias, further fueling doubts about the feasibility of sutureless connector technology.^3^

At the same time, regulatory demands increased. The FDA began shifting from the accessible 510(k) route to full Premarket Approval (PMA) for high-risk vascular devices—a trend formalized by the 2002 MDUFMA and its 2007 update. This raised both costs and complexity, especially for startups. As development grew harder to fund and execute, investor and industry interest waned. Consequently, even technologies showing early promise were discontinued before reaching improved, next-generation versions. Cultural resistance compounded the challenge. Deep-rooted pride in the precision and craftsmanship of hand-sewn anastomoses—and strong accountability for outcomes—made many surgeons reluctant to adopt mechanical alternatives, despite their theoretical appeal.

The exceptions were the PAS-Port and C-Port systems.^4^^,^^5^ These carefully engineered solutions demonstrated non-inferior patency compared to hand-sewn anastomoses and received both CE-mark and FDA approval. The C-Port system accommodated a wide range of distal target vessel diameters—including the smallest graftable coronaries—showing its versatility in that regard. Their most important achievement was proving that sutureless connectors—both proximal and distal—could effectively rival the surgical gold standard. A dedicated endoscopic variant, the C-Port Flex-A, was introduced and showed promise in facilitating TECAB procedures.^6^ Yet despite this progress, adoption remained limited, and both systems were discontinued in 2017 after roughly a decade—ending the availability of the only sutureless coronary anastomotic platforms ever brought to market.

So where does that leave us today? The history of these devices offers important lessons. Despite careful engineering, the C-Port distal device was overly complex and difficult to deploy. Upon retraction, it left a hole in the coronary artery that required manual closure. Its end-to-side geometry limits applicability to single grafts, and endoscopic application could be difficult due to visual obstruction. Finally, manufacturing complexity translated into high costs, rendering the devices prohibitively expensive in many markets. This experience shows that durable patency is essential—but not sufficient. Also required are a simple, user-friendly deployment process; application versatility, meaning compatibility with a full range of vessel sizes and anatomies; and cost-effectiveness. In addition, a wide anastomotic orifice allowing catheter access would be desirable, should future interventions be needed.

Coronary artery bypass grafting is at a crossroads. Patients increasingly demand less invasive options—sometimes choosing PCI against guideline recommendations. Hospitals and insurers aim to reduce costs and staffing burdens, while surgeons seek to offer the least traumatic treatment. Industry momentum is returning, with Intuitive Surgical (Sunnyvale, CA, USA) reentering the cardiac space with its upcoming Gen 5 platform, and other players showing renewed interest. Distal sutureless connector technologies are urgently needed to support the transition toward less invasive, off-pump, and robotic CABG. As the risks of aortic manipulation gain recognition, distal solutions—especially those enabling Y-graft proximal configurations—deserve priority. New connector systems could also help broaden the use of traditional open-chest off-pump procedures, extend their benefits to more patients, and promote greater uptake of arterial grafts by simplifying anastomosis construction. Given past experience, the surgical community should take an active role in shaping next-generation solutions—learning not only from earlier design flaws in patency and intraluminal footprint, but also addressing usability and cost. When well-designed, such systems can shorten hospital stay and recovery, offsetting procedural costs and benefiting patients, providers, and payers alike.

We should not just move beyond past attempts—we must build on them. Smarter, simpler solutions enabled by interdisciplinary collaboration—including surgeons, engineers, and industry.

## Data Availability

No new data were generated or analyzed in support of this research.
